# CLEC4G Promotes Pancreatic Cancer Progression by Suppressing Cathepsin B‐Mediated Ferroptosis: Evidence From Mendelian Randomization Study and Experimental Validation

**DOI:** 10.1155/humu/7359548

**Published:** 2026-06-10

**Authors:** Zhichen Jiang, Pengcheng Ma, Shaobo Zhang, Ze Jin, Yuanyu Wang, Yucheng Zhou, Qicong Zhu, Chao Lu, Ninghui Tu, Zhi′ang Zhang, Yiping Mou, Weiwei Jin

**Affiliations:** ^1^ General Surgery, Cancer Center, Department of Gastrointestinal and Pancreatic Surgery, Zhejiang Provincial People′s Hospital (Affiliated People′s Hospital), Hangzhou Medical College, Hangzhou, Zhejiang, China, hznu.edu.cn; ^2^ Key Laboratory of Gastroenterology of Zhejiang Province, Hangzhou, Zhejiang, China; ^3^ State Key Laboratory of Molecular Oncology, National Cancer Center/National Clinical Research Center for Cancer/Cancer Hospital, Chinese Academy of Medical Sciences and Peking Union Medical College, Beijing, China, cacms.ac.cn

**Keywords:** cathepsin B, CLEC4G, ferroptosis, Mendelian randomization, pancreatic cancer, pQTL

## Abstract

Pancreatic cancer (PC) is highly lethal and lacks causal biomarkers that can inform mechanism‐based therapies. Ferroptosis is an iron‐dependent form of regulated cell death implicated in PC, but upstream determinants of ferroptosis in PC remain unclear. We integrated large‐scale proteomic quantitative trait locus (pQTL) resources with Mendelian randomization (MR) and cell‐based experiments to identify causal regulators of ferroptosis relevant to PC. Using two‐sample MR with plasma pQTL data from the deCODE cohort and the UK Biobank Pharma Proteomics Project and PC genome‐wide association summary statistics from FinnGen, we screened 159 FerrDb‐defined ferroptosis‐related proteins and identified three ferroptosis‐related proteins (CTSB, IDO1, and MDM4) with significant causal effects on PC risk. A proteome‐wide scan further uncovered 13 proteins associated with PC. Two‐step mediation MR supported a causal pathway from CLEC4G through the ferroptosis regulator CTSB to PC risk. In vitro, CLEC4G knockdown increased CTSB expression and ferroptosis activity, which suppressed PC cell proliferation, colony formation, migration, and invasion. Together, our genetic and experimental evidence indicates that CLEC4G promotes PC progression by limiting CTSB‐associated ferroptotic activity in PC cells and supports further investigation of the CLEC4G–CTSB axis in PC.

## 1. Introduction

Pancreatic cancer (PC) is one of the most lethal malignancies, with an extremely poor prognosis—the 5‐year survival rate is only around 13%. Despite some therapeutic advances in recent years, overall outcomes for PC remain very limited [1, 2]. Most patients present at an advanced stage and are no longer eligible for curative surgery, and even with resection plus multiagent chemotherapy, only a small fraction achieve long‐term survival [1–4]. By 2040, PC is projected to become the second leading cause of cancer‐related death worldwide, surpassed only by lung cancer [5].

Ferroptosis, a regulated form of iron‐dependent cell death distinct from apoptosis and other canonical cell death programs, has emerged as a biologically relevant process in cancer progression and treatment response [6–8]. In PC, however, the role of ferroptosis appears to be highly context dependent. Excessive ferroptosis can activate a TMEM173/STING‐dependent DNA‐sensing pathway that accelerates pancreatic tumor growth [9], whereas other evidence supports a tumor‐suppressive role for ferroptosis in PC [6]. These apparently divergent observations suggest that ferroptosis is not simply “beneficial” or “harmful” in PC; rather, its biological consequences may depend on the upstream molecular regulators and cellular context. Therefore, identifying causal regulators that connect ferroptosis to PC risk and malignant behavior is essential for translating ferroptosis biology into therapeutic insight.

Mendelian randomization (MR) provides a useful framework for addressing this question because it leverages the random assortment of single‐nucleotide polymorphisms (SNPs) at conception to reduce confounding and reverse causation in causal inference [10]. MR has been widely applied to identify etiologic factors and therapeutic targets across diseases [11, 12]. For example, MR evidence has supported obesity as a causal risk factor for PC, reinforcing the importance of obesity control in PC prevention [12]. MR has also established a causal relationship between elevated blood cholesterol and coronary heart disease, providing genetic support for lipid‐lowering therapy [13]. With the development of high‐throughput omics technologies, two‐sample MR based on molecular quantitative trait loci has become an increasingly powerful strategy for prioritizing disease‐related biomarkers and drug targets [10].

Among molecular MR approaches, proteomic MR is particularly well suited for target discovery because proteins are closer to cellular function and therapeutic intervention than upstream transcriptomic signals. Protein quantitative trait loci (pQTLs) can therefore provide more direct clues to the causal role of circulating or tissue‐related proteins in disease [14]. Large‐scale proteomic GWAS have identified tens of thousands of pQTL signals, enabling systematic discovery of causal disease factors and therapeutic targets. For instance, Ferkingstad et al. identified more than 18,000 protein‐trait associations in 35,559 Icelanders and proposed 938 potentially druggable causal proteins by integrating these data with disease GWAS results [14]. Similarly, Deng et al. profiled plasma proteins in more than 53,000 individuals, constructed a comprehensive disease‐protein atlas, identified 474 plasma proteins with causal links to diseases, and highlighted 37 potential drug targets with repurposing opportunities [15]. These advances provide a strong rationale for integrating pQTL‐based MR with ferroptosis biology to uncover causal protein networks in PC.

CLEC4G encodes a C‐type lectin receptor predominantly expressed on liver sinusoidal endothelial cells and certain immune cells, where it mediates pathogen recognition and cell adhesion [16, 17]. CLEC4G can promote the adhesion of circulating tumor cells to hepatic sinusoidal endothelium, thereby facilitating colorectal cancer liver metastasis [16]. In breast cancer, tumor‐associated macrophages with high CLEC4G expression bind to the BTN3A3 receptor on tumor cells, enhancing stem‐like properties [18]. However, whether CLEC4G contributes to PC pathogenesis, and whether it acts through ferroptosis‐related mechanisms, remains unknown.

CTSB, in contrast, is a lysosomal cysteine protease involved in protein turnover, degradation, and lysosome‐mediated cell death. Aberrant CTSB activity contributes to the progression of multiple tumors [19]. In PC, CTSB is overexpressed in stem‐like tumor cells and is associated with significantly worse patient prognosis [20]. Immunohistochemical analyses show that 96% of pancreatic tumors express CTSB, and high CTSB levels correlate with higher tumor grade and lymphatic invasion [21]. Additionally, CTSB promotes PC cell invasion and progression by regulating autophagy and metabolic reprogramming [22]. Importantly, recent evidence also implicates CTSB as a critical mediator in the execution of ferroptotic cell death [23]. These observations raise the possibility that CTSB may serve as a mechanistic bridge between upstream protein regulators and ferroptosis‐associated PC biology.

In this study, we employed a multistep strategy combining pQTL‐based MR, mediation MR, and experimental validation to identify causal protein regulators of ferroptosis in PC. Through two‐sample MR and mediation analyses, we identified a causal pathway in which CLEC4G influences PC risk through CTSB‐dependent modulation of ferroptosis. We further validated this axis in vitro, demonstrating that CLEC4G regulates CTSB expression and that perturbing the CLEC4G‐CTSB pathway alters ferroptosis sensitivity and the malignant behavior of PC cells. These findings provide a more integrated understanding of the CLEC4G–CTSB‐ferroptosis axis in PC development and support its potential as a therapeutic target.

## 2. Methods

### 2.1. Study Design

**Figure 1:**
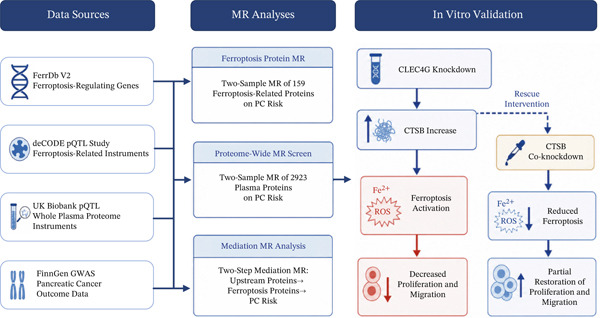
Overview of the multistage study design and workflow. A ferroptosis regulator gene set from FerrDb defined candidate ferroptosis‐related proteins for an initial pQTL‐based Mendelian randomization (MR) screen (Stage 1) in the deCODE cohort, which identified ferroptosis‐associated proteins linked to pancreatic cancer risk. This was followed by a proteome‐wide MR analysis (Stage 2) in the UK Biobank Pharma Proteomics Project (UKB‐PPP), uncovering additional upstream protein candidates associated with pancreatic cancer. A two‐step mediation MR framework was then applied to test causal pathways where an upstream protein influences pancreatic cancer through a ferroptosis‐related mediator, ultimately highlighting the CLEC4G–CTSB protein axis. Finally, this pathway was validated experimentally: silencing CLEC4G in pancreatic cancer cells increased CTSB expression and activated ferroptosis, accompanied by reduced tumor cell growth and invasion, whereas CTSB inhibition reversed these changes observed upon CLEC4G knockdown.

This study was designed and reported in accordance with the STROBE‐MR guideline for MR studies [24] (Table S1). A multistage two‐sample MR framework was implemented to (i) systematically test ferroptosis‐related proteins for causal effects on PC risk, (ii) perform a proteome‐wide causal screen to identify upstream candidate proteins associated with PC, and (iii) construct and quantify an upstream protein, ferroptosis‐related protein, PC causal chain using a two‐step mediation MR. We further conducted in vitro experiments to validate the key MR‐identified axis at the cellular level (Figure 1).

All analyses were performed using publicly available summary statistics; the original studies obtained informed consent and ethical approval, and no additional ethics approval was required for this secondary analysis.

### 2.2. Data Sources

#### 2.2.1. Ferroptosis‐Related Gene Set

A curated list of ferroptosis‐regulating genes was obtained from FerrDb V2 [25]. FerrDb V2 contains 1001 ferroptosis‐related molecules, including 621 regulator genes (e.g., drivers and suppressors). These regulator genes were used to define the candidate ferroptosis‐related proteins tested in Stage 1.

#### 2.2.2. pQTL Datasets

##### 2.2.2.1. Stage 1 (Ferroptosis‐Related Proteins)

Genetic association summary statistics for plasma protein levels were extracted from the Icelandic deCODE plasma proteome GWAS [14], which measured 4900 plasma proteins in 35,559 participants and identified extensive pQTL signals.

##### 2.2.2.2. Stage 2 (Proteome‐Wide Screening and Upstream Exposures)

For the proteome‐wide MR screen, we used pQTL summary data from the UK Biobank Pharma Proteomics Project (UKB‐PPP) [26], which quantified 2923 plasma proteins in 54,219 participants of European ancestry using an Olink platform.

#### 2.2.3. Outcome GWAS

PC GWAS summary statistics were obtained from FinnGen release R12 [27], comprising approximately 3100 PC cases and 378,000 controls of European descent. The FinnGen study is a large‐scale genomics initiative that has analyzed over 500,000 Finnish biobank samples and correlated genetic variation with health data to understand disease mechanisms and predispositions.

All source studies were predominantly of European ancestry, reducing potential population stratification across two‐sample MR.

RNA sequencing data and mutation data for PAAD were obtained from the TCGA database.

### 2.3. Instrumental Variable (IVs) Selection

IVs were selected from exposure pQTL datasets following a consistent set of criteria across analyses. For each protein, we initially chose SNPs associated at genome‐wide significance (p < 5 × 10^−8^). To ensure independence, the SNPs were clumped for linkage disequilibrium (LD) using the 1000 Genomes European reference (r^2^ < 0.001 within a 10 Mb window), retaining only the most significant variant from each LD block. We prioritized cis‐acting pQTLs located within ±1 Mb of the encoding gene and avoided trans‐acting instruments when possible to minimize horizontal pleiotropy. Palindromic SNPs with ambiguous strands were excluded or resolved using allele frequency information. Instrument strength was assessed using F‐statistics to minimize weak‐instrument bias. For each exposure, the F‐statistic was calculated as *F* = *R* ^2^(*n* − *k* − 1)/[*k* (1 − *R* ^2^)], where *R* ^2^ represents the proportion of exposure variance explained by the selected genetic instruments, *n* is the sample size of the exposure pQTL dataset, and *k* is the number of instruments. An F‐statistic > 10 was considered indicative of sufficient instrument strength, whereas instruments with F‐statistics < 10 were regarded as weak instruments and excluded from subsequent MR analyses [28]. In the context of two‐step mediation MR, we ensured that the IV sets for the upstream protein (Step 1) and the ferroptosis‐related mediator protein (Step 2) did not overlap, maintaining independence of instruments across the two steps.

### 2.4. Two‐Sample MR Analysis

Two‐Sample MR analyses were conducted to estimate the causal effects of genetically proxied protein levels on PC risk. Multiple MR methods were employed, including inverse variance weighted (IVW) as the primary approach [29], as well as weighted median (WM) [30], MR‐Egger regression [31], weighted mode (WMO), and simple mode (SMO). The IVW method was used as the primary estimator because it provides the most statistically efficient estimate when all genetic instruments are valid or when horizontal pleiotropic effects are balanced [29, 32]. To examine the robustness of the IVW results under potential violations of MR assumptions, several complementary estimators were applied. The WM method was used because it can provide a consistent causal estimate when at least 50% of the total instrument weight is contributed by valid variants [30]. MR‐Egger regression was used to detect and adjust for potential directional horizontal pleiotropy, with the intercept term serving as an indicator of unbalanced pleiotropic effects [31].WMO and SMO estimators were additionally applied as sensitivity methods, as they can provide valid inference when the largest cluster of instruments with similar causal estimates represents valid instruments [33].

Heterogeneity among instrument‐specific causal estimates was assessed using Cochran′s Q test, with *p* < 0.05 indicating significant heterogeneity. Horizontal pleiotropy was evaluated using the MR‐Egger intercept test and MR‐PRESSO. MR‐PRESSO was used to detect horizontal pleiotropic outliers and, when present, to generate outlier‐corrected estimates [34]. Leave‐one‐out analyses were further performed to determine whether the causal estimates were driven by any single variant.

### 2.5. Two‐Step Mediation MR

To investigate whether an upstream protein influences PC through a ferroptosis‐related mediator, we performed a two‐step mediation MR analysis following established methods for causal mediation with genetic instruments [35]. This framework posits a causal chain in which genetic variants affect an upstream protein (exposure), the upstream protein affects a mediator protein, and the mediator in turn affects the disease outcome. For Step 1 of the mediation analysis, we selected instruments for the upstream protein from the UKB‐PPP pQTL data and obtained the SNP‐to‐mediator association estimates from the deCODE pQTL dataset. We then conducted MR to estimate the causal effect of the upstream protein on the mediator′s level. For Step 2, we identified instruments for the mediator protein from the deCODE pQTL data and retrieved the SNP‐to‐outcome (PC) association estimates from FinnGen R12, then performed MR to estimate the effect of the mediator protein on PC risk. If both MR steps provided evidence supporting causality (i.e., the upstream protein affects the mediator, and the mediator affects the outcome), we inferred the presence of an indirect causal pathway. We quantified the indirect effect of the upstream protein on PC mediated through the ferroptosis‐related protein by multiplying the MR estimate from Step 1 (upstream → mediator) by the MR estimate from Step 2 (mediator → outcome). The proportion of the upstream protein′s total effect on PC that is mediated by the ferroptosis‐related protein was then calculated as the ratio of this indirect effect to the total effect (as estimated by a one‐step MR of the upstream protein on PC). Confidence intervals for the indirect effect and mediation proportion were derived using the delta method. Throughout the mediation MR, we took care to use independent instrument sets for each step and considered the results significant only if both steps yielded consistent evidence of causality.

### 2.6. Relationship of CLEC4G With Somatic Mutation Landscape

Somatic mutation profiles (MAF format) of the TCGA pancreatic adenocarcinoma cohort (TCGA‐PAAD) were obtained from the TCGA‐Genomic Data Commons repository. Matched transcriptomic data from the same cohort were retrieved, and only samples with both mutation and CLEC4G expression information were included for downstream analyses. Patients were dichotomized into CLEC4G‐high (*n* = 83) and CLEC4G‐low (*n* = 79) groups using the median CLEC4G expression as the cutoff. Gene‐level alteration status of CLEC4G was queried in the TCGA‐PAAD cohort using cBioPortal. Mutation annotation and visualization were performed in R (Version 4.5.2) using the maftools package.

### 2.7. Cell Culture

PC cells AsPC‐1, MIA PaCa‐2, BxPC‐3, Panc‐1, and SW1990 were purchased from the American Type Culture Collection (ATCC). AsPC‐1 and BxPC‐3 cells were cultured in RPMI‐1640 medium (XiGong) supplemented with 10% fetal bovine serum (XiGong) and penicillin (100 U/mL), streptomycin (100 mg/mL). Panc‐1 and SW1990 cells were cultured in DMEM medium (XiGong) supplemented with 10% fetal bovine serum, penicillin (100 U/mL), and streptomycin (100 mg/mL). MIA PaCa‐2 cells were cultured in DMEM medium with 10% fetal bovine serum (XiGong), 2.5% horse serum and penicillin (100 U/mL), streptomycin (100 mg/mL).

### 2.8. siRNA Transfection

At 40%–60% confluence, cells were transfected using Lipofectamine RNAiMAX (Invitrogen) according to the manufacturer′s instructions. Briefly, specific siRNA or negative control siRNA (GenePharma) was mixed with the transfection reagent in Opti‐MEM medium (Gibco) to form complexes, which were then added to the cells. After 6 h, the medium was replaced with fresh complete medium. Knockdown efficiency was evaluated by western blotting 48–72 h after transfection.

### 2.9. Western Blot Analysis

The medium was discarded, and cells were washed with PBS, scraped, and collected. After lysis with RIPA buffer on ice for 30 min, the samples were centrifuged at 13000 g for 20 min at 4°C. The protein supernatant was quantified by BCA assay, mixed with loading buffer and reducing agent to 1 *μ*g/*μ*L, and boiled at 98°C for 10 min. Following SDS‐PAGE and transfer, the membrane was blocked with 5% skim milk, incubated overnight at 4°C with primary antibodies against LSECtin (Abcam, ab181196), CTSB (Proteintech, 12216‐1‐AP), GAPDH (Proteintech, 80570‐1‐RR), washed, and then incubated with HRP‐conjugated secondary antibody for 2 h at room temperature. Protein bands were finally visualized using a qTouch Western Blot Imager (RWD) with ECL reagent.

### 2.10. RT‐qPCR

Total RNA was extracted from cells using TRIzol reagent according to the manufacturer′s instructions. RNA concentration and purity were assessed using a spectrophotometer. Complementary DNA was synthesized using a reverse transcription kit, and quantitative real‐time PCR was performed using SYBR Green‐based detection. Relative CTSB mRNA expression was normalized to GAPDH and calculated using the 2−*ΔΔ*Ct method. Primer sequences are listed in Table S2.

### 2.11. Flow Cytometry

Intracellular Fe^2+^ and ROS were detected using the Ferroptosis Fluorometric Assay Kit with RhoNox‐6 and CM‐H2DCFDA (Beyotime, C1190S) according to the manufacturer′s instructions. For intracellular Fe^2+^ detection, adherent cells were digested with trypsin, resuspended, and washed three times with serum‐free medium. The cells were then incubated with RhoNox‐6 working solution at 37°C for 30 min. Similarly, intracellular ROS was labeled using the fluorescent probe CM‐H2DCFDA. Stained samples were analyzed on a CytoFLEX S flow cytometer (Beckman Coulter), and data were processed using FlowJo software.

### 2.12. Cellular Immunofluorescence Staining

Cells were seeded in a 24‐well plate. The RhoNox‐6/CM‐H2DCFDA Staining Solution (Beyotime, C1190S) working solution was prepared. When the cells reached approximately 50% confluence, the culture medium was removed and replaced with the working solution, followed by incubation at 37°C for 30 min. Subsequently, Hoechst 33258 (MCE) staining solution was added and incubated for 10 min to label the nuclei. Finally, the cells were observed and images were captured using a fluorescent confocal microscope.

### 2.13. Cell Proliferation Assays

Cells were digested from the dish with trypsin and prepared with medium with 10% FBS. Two thousand cells in each well with a volume of 100 *μ*L were plated in 96‐well plates. Cell proliferation was measured using CCK‐8 reagent at hours 0, 24, 48, and 72. Specifically, CCK‐8 reagent was added to the cell culture medium at a ratio of 1:10, and the absorbance was measured at 450 nm after incubation for 1 h at 37°C. The absorbance at Day 0 was designated as 1. The relative cell proliferation rate at each time point was calculated.

### 2.14. Cell Colony Formation Assay

Cells were seeded in 6‐well plates, 1000–2000 cells per well, and allowed to grow in complete medium for 7–14 days, with the medium refreshed every 3 days. Once visible colonies had formed, the medium was removed. The cells were washed twice with PBS, fixed with 4% paraformaldehyde for 20 min at room temperature, and then stained with 0.1% crystal violet solution for 30 min. After staining, the plates were gently rinsed with tap water and air‐dried overnight. Colonies were counted manually or analyzed using ImageJ.

### 2.15. Cell Migration and Invasion Assay

AsPC‐1, MIA PaCa‐2, or BxPC‐3 cells (1.5–3.0 × 10^5^ cells per well) were seeded into the upper chambers of 24‐well Transwell plates (8 *μ*m pore size; Corning, New York, United States) and allowed to migrate or invade for 24–48 h. Thereafter, the inserts were fixed and stained with 0.5% crystal violet in methanol for 2 h. The number of migrated/invaded cells was quantified by counting three randomly selected fields per insert under a microscope. Images were acquired using a NIKON Eclipse Ti‐S microscope.

### 2.16. Statistical Analysis

All R‐based analyses were performed and rechecked in R (Version 4.5.2), and all reported results and visualizations were confirmed to be reproducible under this updated software environment. Specifically, MR analyses, including the Stage 1 ferroptosis‐focused MR screen, Stage 2 proteome‐wide MR screen, two‐step mediation MR, heterogeneity assessment, pleiotropy assessment, and leave‐one‐out sensitivity analyses, were performed using the Two SampleMR package and related MR packages. The TCGA‐PAAD mutation annotation and visualization analyses were performed using the maftools package. For the two discovery‐stage MR screens, multiple testing was controlled using Bonferroni correction according to the number of proteins tested in each stage. Specifically, the significance threshold was set at *p* < 0.05/159 = 3.14 × 10^−4^ for the Stage 1 ferroptosis‐focused MR screen and *p* < 0.05/2619 = 1.91 × 10^−5^ for the Stage 2 proteome‐wide MR screen. For hypothesis‐driven follow‐up analyses, including specific mediation analyses and experimental validation, two‐tailed *p* < 0.05 was considered statistically significant. Effect estimates are reported with 95% confidence intervals. Experimental data were analyzed using GraphPad Prism.

## 3. Result

### 3.1. Causal Effects of Ferroptosis‐Related Proteins on PC Risk

**Figure 2:**
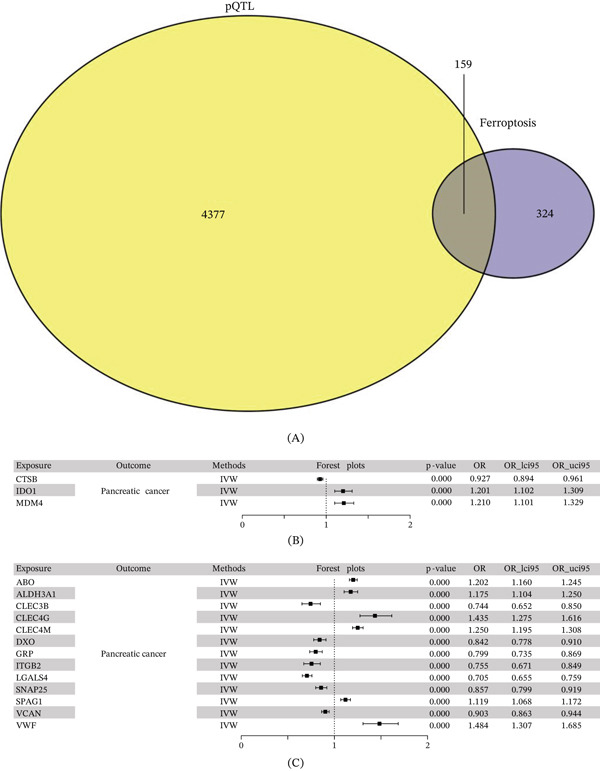
Two‐sample Mendelian randomization (MR) screening identifies ferroptosis‐related and proteome‐wide causal proteins for pancreatic cancer (PC). (A) Venn diagram showing the overlap between proteins with available pQTL instruments and a curated ferroptosis regulator gene set (FerrDb v2). Out of 4900 plasma proteins with genetic instruments from the Icelandic deCODE study (35,559 participants) and 621 ferroptosis‐related genes from FerrDb, 159 overlapping ferroptosis‐related proteins were retained for downstream MR analysis. (B) Forest plot summarizing the causal effects of the three ferroptosis‐related proteins that remained significantly associated with PC risk after Bonferroni correction in the ferroptosis‐focused MR screen. (C) Forest plot of the top hits from the proteome‐wide MR scan using UKB‐PPP pQTL data (*n* = 54,219) as exposures and FinnGen R12 pancreatic cancer as the outcome

Using ferroptosis regulators curated from FerrDb V2 and plasma pQTL summary statistics from the deCODE cohort, we mapped ferroptosis‐related genes to measurable proteins and retained 159 proteins with valid genetic instruments for downstream causal inference (Figure 2A). We then conducted two‐sample MR to assess whether genetically predicted levels of these ferroptosis‐related proteins were causally associated with PC risk. After Bonferroni correction for 159 ferroptosis‐related proteins (significance threshold *p* < 3.14 × 10^−4^), we identified three ferroptosis‐related proteins with Bonferroni‐significant causal associations with PC risk. Specifically, genetically predicted higher levels of CTSB were associated with a lower risk of PC (IVW OR = 0.93, 95% CI 0.89–0.96, *p* = 3.5 × 10^−5^). In contrast, higher genetically predicted IDO1 levels were associated with an increased risk of PC (IVW OR = 1.20, 95% CI 1.10–1.31, *p* = 3.23 × 10^−5^). Similarly, elevated MDM4 levels showed a positive association with PC risk (IVW OR = 1.21, 95% CI 1.10–1.33, *p* = 7.03 × 10^−5^). These findings were consistent across sensitivity analyses: the WM MR method yielded significant results for each protein, and no evidence of horizontal pleiotropy was detected (Figure 2B) (Tables S3 and S4).

### 3.2. Proteome‐Wide MR Screen for Upstream PC‐Associated Proteins

Leveraging large‐scale pQTL data from the UKB‐PPP, we tested the causal relevance of 2619 plasma proteins for PC risk. After Bonferroni correction (significance threshold *p* < 1.91 × 10^−5^), 29 proteins showed Bonferroni‐significant associations with PC risk (Table S5). After excluding associations with evidence of horizontal pleiotropy or heterogeneity, 13 proteins remained robustly associated with PC risk. Among these, genetically predicted higher levels of six proteins (ABO, ALDH3A1, CLEC4G, CLEC4M, SPAG1, and VWF) were associated with increased PC risk, whereas seven proteins (CLEC3B, DXO, GRP, ITGB2, LGALS4, SNAP25, and VCAN) conferred a protective effect (Figure 2C).

### 3.3. Two‐Step Mediation MR Analysis

**Figure 3:**
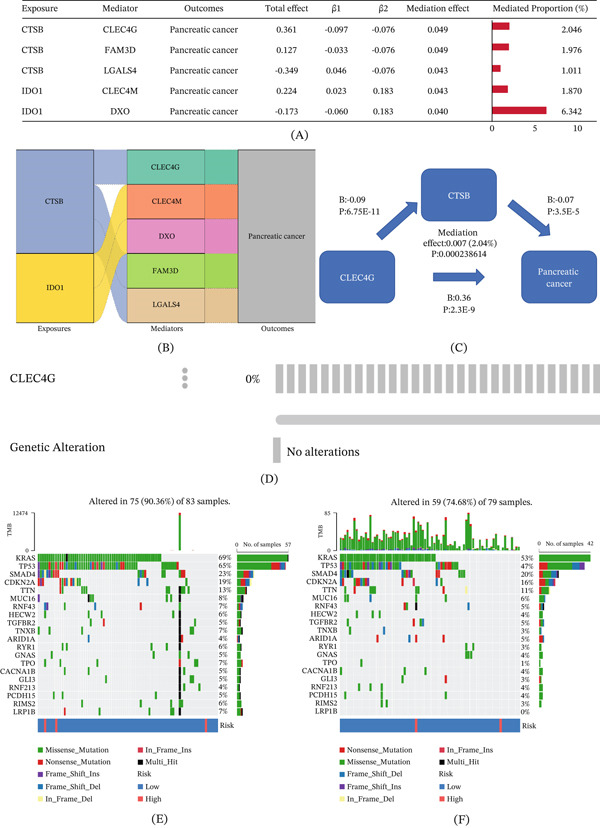
Two‐step MR‐based mediation analysis linking upstream proteins, ferroptosis regulators, and pancreatic cancer (PC) risk and relationship of CLEC4G expression with the somatic mutation landscape in PC. (A) Five upstream protein–ferroptosis regulator pairs exhibit significant mediated effects on PC risk. (B) Sankey diagram visualizing the exposure–mediator–outcome relationships identified by two‐step mediation MR. (C) Analysis of the CLEC4G–CTSB pathway demonstrates that CLEC4G′s effect on PC risk is largely mediated via CTSB. (D) Gene‐level alteration frequency of CLEC4G in the TCGA pancreatic adenocarcinoma cohort (TCGA‐PAAD) queried from cBioPortal. (E) Somatic mutation landscape (oncoplot) of the CLEC4G‐high group (*n* = 83). (F) Somatic mutation landscape (oncoplot) of the CLEC4G‐low group (*n* = 79).

We next investigated whether the effects of the above 13 upstream proteins on PC are mediated through ferroptosis‐related pathways. A two‐step MR mediation analysis identified five putative causal chains of the form “upstream → ferroptosis regulator → PC” with significant mediation effects (Figure 3A,B). Among these, the CLEC4G→CTSB→PC axis stood out as the most significant. This result indicates that the deleterious effect of CLEC4G on PC risk is largely mediated by its influence on CTSB (Figure 3C).

### 3.4. CLEC4G‐High Tumors Display a Driver‐Mutation–Enriched Somatic Landscape in PC

Using the TCGA‐PAAD cohort, we first queried gene‐level alterations of CLEC4G and observed that CLEC4G is rarely altered in PC (Figure 3D). We then stratified patients into CLEC4G‐high (*n* = 83) and CLEC4G‐low (*n* = 79) groups based on CLEC4G expression and generated mutation landscape plots for each group (Figure 3E,F). Notably, KRAS and TP53 mutations were more frequent in the CLEC4G‐high group (KRAS: 69% vs. 53%; TP53: 65% vs. 47%), suggesting an association between CLEC4G‐high tumors and a driver‐mutation–enriched genomic context.

### 3.5. In Vitro Experimental Validation

**Figure 4:**
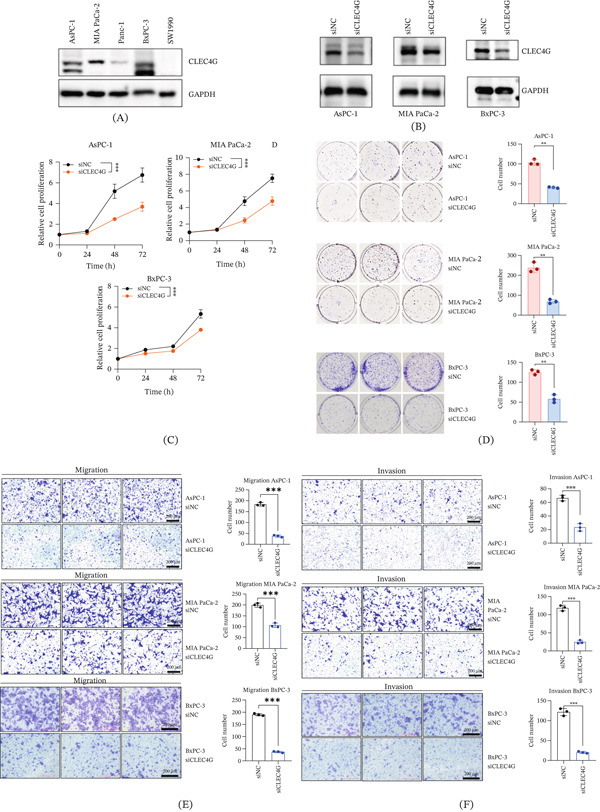
CLEC4G expression in pancreatic cancer cell lines and the effects of CLEC4G knockdown on malignant phenotypes. (A) Basal CLEC4G protein levels in the indicated pancreatic cancer cell lines were assessed by western blotting. AsPC‐1, MIA PaCa‐2, and BxPC‐3 cells showed relatively high CLEC4G expression and were selected for subsequent functional analyses. (B) Knockdown efficiency of CLEC4G in AsPC‐1, MIA PaCa‐2, and BxPC‐3 cells was confirmed by western blotting after siRNA transfection. (C) Cell proliferation curves of AsPC‐1, MIA PaCa‐2, and BxPC‐3 cells transfected with control siRNA (siNC) or siRNA targeting CLEC4G (siCLEC4G). (D) Colony formation assays of AsPC‐1, MIA PaCa‐2, and BxPC‐3 cells following CLEC4G knockdown. (E) Transwell migration assays of AsPC‐1, MIA PaCa‐2, and BxPC‐3 cells after CLEC4G silencing (Scale − bars = 200 *μ*m). (F) Transwell invasion assays of AsPC‐1, MIA PaCa‐2, and BxPC‐3 cells after CLEC4G silencing (Scale − bars = 50 *μ*m). Data are presented as mean ± SD. ∗∗*p* < 0.01, ∗∗∗*p* < 0.001.

To validate the CLEC4G‐CTSB‐ferroptosis axis suggested by MR, we performed functional experiments in PC cell lines. Western blot analysis across five human PC cell lines showed that CLEC4G was highly expressed in AsPC‐1, MIA PaCa‐2, and BxPC‐3 cells (Figures 4A and S1A). Therefore, these three cell lines were selected for subsequent loss‐of‐function analyses. CLEC4G knockdown efficiency was confirmed by western blotting in AsPC‐1, MIA PaCa‐2, and BxPC‐3 cells (Figures 4B and S1B). In these three cell lines, CLEC4G silencing markedly inhibited cell proliferation (Figure 4C), colony formation (Figure 4D), migration (Figure 4E), and invasion (Figure 4F). These findings further support a protumor role of CLEC4G in PC and extend the consistency of the in vitro results across multiple CLEC4G‐high cell lines.

**Figure 5:**
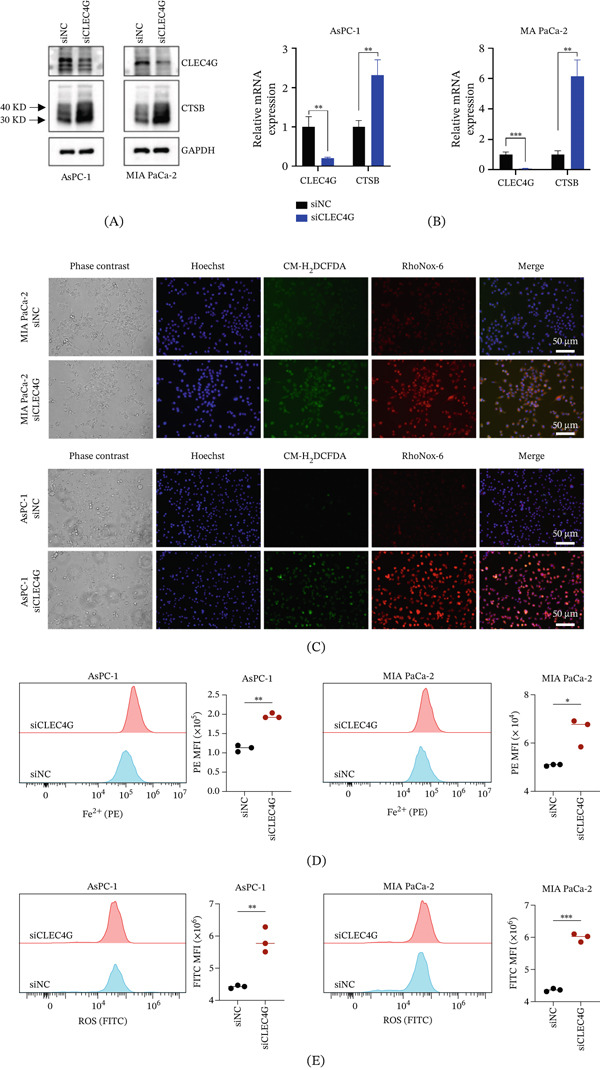
CLEC4G knockdown increases CTSB expression and enhances ferroptosis‐associated changes in pancreatic cancer cells. (A) Western blot analysis of CLEC4G and CTSB in MIA PaCa‐2 and AsPC‐1 cells after transfection with siNC or siCLEC4G. Total CTSB was quantified as the combined densitometric intensity of pro‐CTSB and mature CTSB normalized to the loading control. (B) RT‐qPCR analysis of CTSB mRNA expression after CLEC4G knockdown. (C) Fluorescence imaging of intracellular oxidative stress and labile iron‐associated signals in MIA PaCa‐2 and AsPC‐1 cells after CLEC4G knockdown. Scale bars = 50 *μ*m. (D, E) Flow cytometry profiles and quantification of labile Fe^2+^ and ROS‐associated fluorescence in AsPC‐1 and MIA PaCa‐2 cells transfected with siNC or siCLEC4G. Data are presented as mean ± SD. ∗*p* < 0.05, ∗∗*p* < 0.01, ∗∗∗*p* < 0.001.

We next examined whether CLEC4G knockdown affected CTSB expression and ferroptosis‐associated activity. CLEC4G silencing led to a marked increase in CTSB protein levels in both AsPC‐1 and MIA PaCa‐2 cells (Figure 5A). Densitometric quantification of the western blot bands after normalization to GAPDH, together with RT‐qPCR analysis, further confirmed the upregulation of CTSB following CLEC4G knockdown (Figures 5B and S1C). In parallel, intracellular Fe2+ and lipid ROS levels were also increased after CLEC4G depletion (Figure 5C–E). Collectively, these findings support enhanced CTSB‐associated ferroptosis activity after CLEC4G knockdown.

**Figure 6:**
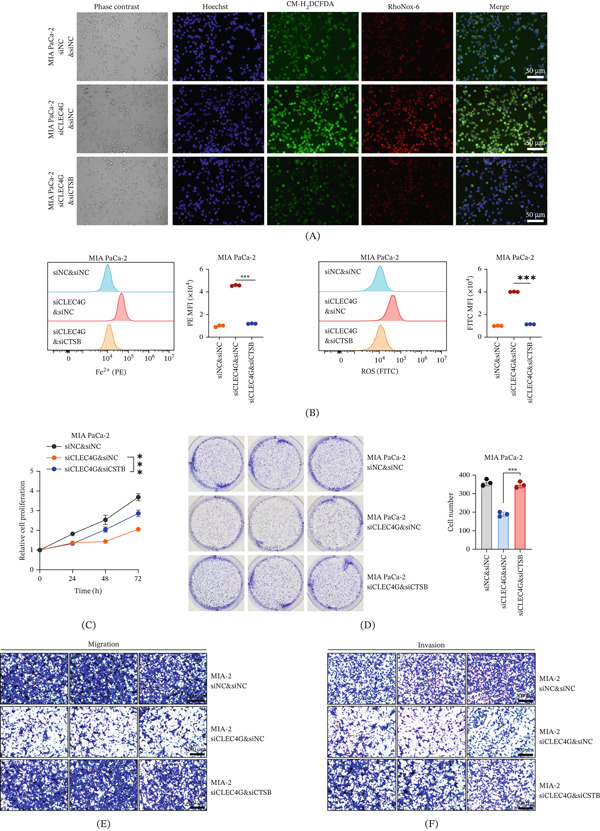
Inhibition of CTSB reverses CLEC4G knockdown‐induced ferroptosis and suppressive effects on tumor cell growth and motility. (A) Flow cytometry measurement of intracellular labile Fe^2+^ in AsPC‐1 pancreatic cancer cells transfected with control siRNA (siNC), CLEC4G siRNA (siCLEC4G), or combined CLEC4G and CTSB siRNAs (siCLEC4G + siCTSB). CLEC4G knockdown increases Fe^2+^ levels compared with control, whereas concurrent CTSB knockdown reduces Fe^2+^ levels toward control (Scale − bars = 50 *μ*m). (B) Flow cytometry measurement of lipid reactive oxygen species (ROS) under the same conditions. CLEC4G knockdown elevates lipid ROS relative to control, and CTSB coknockdown mitigates this increase, returning ROS levels closer to control. (C) Cell proliferation assay (72‐h viability) under the three treatment conditions. CLEC4G knockdown alone reduces cell proliferation compared with control, whereas coknockdown of CTSB leads to higher proliferation than CLEC4G knockdown alone (partial restoration toward control levels). (D) Colony formation assay for AsPC‐1 cells under the three treatments. CLEC4G knockdown yields fewer and smaller colonies than control; in contrast, dual knockdown results in more colonies than CLEC4G knockdown alone. (E) Cell migration assay. CLEC4G knockdown decreases the number of migrating cells relative to control, whereas adding CTSB knockdown increases cell migration compared with CLEC4G knockdown alone. (F) Cell invasion assay (Scale − bars = 200 *μ*m). CLEC4G knockdown reduces cell invasiveness, and dual knockdown of CLEC4G + CTSB leads to greater invasion than CLEC4G knockdown alone (Scale − bars = 200 *μ*m).

To further validate the regulatory mechanism of the CLEC4G–CTSB–ferroptosis axis, we performed rescue experiments. Inhibition of CTSB significantly attenuated the upregulation of ferroptosis induced by CLEC4G knockdown (Figure 6A,B). This result confirms that the increased ferroptosis level caused by CLEC4G suppression is mediated by CTSB. Further examination of malignant phenotypes in PC cells showed that additional inhibition of CTSB following CLEC4G knockdown restored cellular proliferation, colony formation, as well as migratory and invasive capacities (Figure 6C–F). These results are consistent with the genetic findings—reduction of CLEC4G (which elevates CTSB and ferroptosis) led to diminished tumor cell growth and invasiveness in vitro, supporting a functional role for the CLEC4G–CTSB–ferroptosis axis in PC.

## 4. Discussion

In this study, we performed the first systematic MR analysis in PC based on large‐scale pQTL data to identify regulators of ferroptosis. This analysis identified CLEC4G as a novel causal driver of PC that likely promotes tumor progression by constraining a CTSB‐associated ferroptosis response. Using a two‐step MR approach combined with in vitro experiments, we provided the first direct evidence for the involvement of the CLEC4G–CTSB–ferroptosis pathway in PC pathogenesis. These findings provide new insights into ferroptosis regulation in PC and highlight the CLEC4G–CTSB–ferroptosis pathway as a potential target for developing novel targeted therapies.

CLEC4G is an important immune lectin primarily expressed on certain immune cells and endothelial cells. Previous studies have implicated CLEC4G in tumor immune evasion and metastasis. For example, in colorectal cancer, CLEC4G expression promotes hepatic metastasis, and knockout or blockade of CLEC4G significantly reduces liver metastases of colorectal cancer cells [16]. In breast cancer, tumor‐associated macrophages expressing CLEC4G bind to the BTN3A3 receptor on tumor cells, enhancing the stem‐like properties of the cancer cells [18]. A recent study in gastric cancer further showed that CLEC4G can downregulate STAT1 via a circFBXL4/miR‐146a‐5p/STAT1 axis, leading to upregulation of FN1 and CHD4 and thereby promoting cancer cell adhesion, invasion, and lymphatic metastasis [36]. Collectively, these findings establish CLEC4G as an immunomodulatory factor that shapes a tumor‐promoting microenvironment and increases malignant phenotypes. However, prior to our investigation, the role of CLEC4G in PC had not been reported. Here, we found that CLEC4G knockdown increased CTSB expression and was accompanied by enhanced ferroptosis in tumor cells. This discovery reveals a novel link between an immune lectin and the ferroptosis mechanism, extending the functional significance of CLEC4G into the realm of cell death regulation.

CTSB is a lysosomal protease known to promote tumor progression in multiple cancers, including PC. Previous studies reported that CTSB is highly expressed in PC tissues, especially in the stem‐like subpopulation of tumor cells, and that elevated CTSB levels are significantly associated with worse patient prognosis [20]. One mechanism by which CTSB drives tumor invasion and progression is through sustaining autophagy and reprogramming cellular metabolism to support tumor cell survival and aggressiveness. For instance, Cystatin B has been shown to maintain the proteolytic activity of CTSB, thereby enhancing autophagic flux in PC cells and promoting metabolic reprogramming, such as increased glycolysis, ultimately fueling tumor progression [22]. Recent evidence has also linked CTSB to the execution of ferroptosis. CTSB was identified as a critical executioner of ferroptosis: when lipid peroxidation triggers lysosomal membrane permeabilization, CTSB leaks into the cytosol or nucleus, where it degrades structural proteins such as histone H3 and precipitates cell demise. Inhibition of CTSB can markedly rescue the loss of membrane integrity and chromatin degradation caused by ferroptosis, indicating that CTSB mediates downstream execution of ferroptosis signals [23]. In our experimental system, silencing CLEC4G increased CTSB expression, intracellular labile iron, and lipid peroxidation, whereas CTSB coknockdown attenuated these ferroptosis‐associated changes and partially restored malignant phenotypes. Therefore, our findings should not be interpreted as indicating that CTSB is uniformly tumor suppressive in PC. Rather, they support a context‐dependent model in which CTSB may promote tumor progression under some biological conditions, but under CLEC4G‐suppressed conditions is functionally coupled to ferroptosis‐associated execution and reduced malignant phenotypes. Several biologically plausible mechanisms may link a membrane lectin such as CLEC4G to a lysosomal protease such as CTSB. C‐type lectin receptors can undergo internalization and endosomal recycling, and studies of related lectin receptors have shown that their trafficking to endolysosomal compartments can influence downstream signaling, phagosome maturation, and reactive oxygen responses [37–39]. In parallel, lysosome trafficking is known to modulate protease secretion and invasive behavior in tumor cells [40]. Therefore, one possible explanation is that CLEC4G may influence CTSB‐associated ferroptotic and malignant phenotypes by altering lysosome‐related trafficking or signaling states rather than acting solely as a linear regulator of CTSB expression. Although this possibility remains to be tested directly, it provides a biologically reasonable framework for interpreting the CLEC4G‐CTSB association observed in the present study.

Traditional MR studies typically focus on the causal effect of a single exposure on an outcome, and it is often challenging to validate such findings experimentally [12, 32, 41]. Here, we combined a proteome‐wide MR screen, two‐step mediation MR analysis, and biological validation. Leveraging large pQTL resources (deCODE and UKB‐PPP), we conducted an MR scan across the plasma proteome and then used mediation analysis to connect the results, revealing that CTSB (a ferroptosis‐related protein) is a significant mediator of CLEC4G′s effect on PC risk. Finally, we confirmed the genetically predicted pathway through in vitro cell‐based experiments. By integrating genetic epidemiology with molecular biology, our study design transcended the one‐dimensional scope of traditional MR and constructed a multistep causal mechanism from genotype to phenotype. This comprehensive strategy provides a paradigm for elucidating complex, multistage disease mechanisms. Beyond its mechanistic significance, the CLEC4G–CTSB axis may also have translational relevance in PC. This possibility should first be evaluated in clinically relevant PC models, including patient‐derived organoids and orthotopic or patient‐derived xenografts, which are increasingly used to assess therapeutic response in PC [42, 43]. Given the reported links between ferroptosis and gemcitabine sensitivity in PC, as well as the physiological and immune‐context‐dependent expression pattern of CLEC4G/LSECtin, further studies are needed to define whether this axis has biomarker value or therapeutic relevance in specific clinical settings [18, 44].

Despite its strengths, our study has several limitations. First, the MR analyses were based on available summary statistics, and the GWAS and pQTL datasets we used were largely derived from European populations using plasma samples. Population structure bias or dataset‐specific biases may affect the generalizability and reliability of our findings. We attempted to minimize bias through multiple sensitivity analyses, but residual horizontal pleiotropy or confounding could still be present. Second, our functional experiments were performed in vitro using PC cell lines. Although these assays support the MR findings, cell line models cannot fully recapitulate the complexity of the in vivo tumor microenvironment. Third, our mediation MR assumes a linear causal chain in which CLEC4G affects PC risk through CTSB. However, CLEC4G may also influence PC biology through additional intermediaries or parallel pathways, including other ferroptosis regulators, immune‐related mechanisms, or microenvironmental components that were not captured in our analysis. Finally, the precise biochemical mechanism linking CLEC4G to CTSB‐associated ferroptosis activity remains unclear. Further studies using in vivo models, clinically annotated cohorts, and preferably cell‐type‐resolved data will be needed to determine whether the CLEC4G‐CTSB‐ferroptosis axis has prognostic or predictive value in specific patient subgroups.

## 5. Conclusion

This study demonstrates a strategic multiomics approach to identify causal drivers in PC. We identified CLEC4G as a causal factor associated with PC risk and showed that CLEC4G silencing is accompanied by increased CTSB expression and enhanced ferroptosis‐associated activity, thereby suppressing malignant phenotypes in PC cells. Targeting the CLEC4G–CTSB–ferroptosis axis may represent a novel therapeutic strategy against PC.

## Author Contributions

Zhichen Jiang and Pengcheng Ma contributed equally as cofirst authors. Zhichen Jiang and Shaobo Zhang conceived the study, designed the Mendelian randomization (MR) analyses, and interpreted the genetic data. Zhichen Jiang performed the MR analyses with support from Shaobo Zhang. Pengcheng Ma designed and carried out the cell‐based experiments, with assistance from Yuanyu Wang, Yucheng Zhou, Chao Lu, Qicong Zhu, Ninghui Tu; Zhi′ang Zhang in data collection and laboratory work. Zhichen Jiang and Pengcheng Ma drafted the manuscript. Yiping Mou and Weiwei Jin supervised the overall project and provided critical revisions to the manuscript. All authors contributed to data interpretation.

## Funding

This study was supported by the Scientific research fund of national health commission of China, Key health science and technology program of Zhejiang Province (WKJ‐ZJ‐2201); General Scientific Research Project of the Education Department of Zhejiang Province (Y202249378); and Key Project of social welfare program of Zhejiang Science and Technology Department, “Lingyan” Program (2022C03099).

## Disclosure

All authors read and approved the final manuscript.

## Ethics Statement

This study was conducted using publicly available summary‐level genetic/proteomic data and experiments in established cell lines. Ethical approval and informed consent were obtained in the original studies from which the summary statistics were derived. No new human participants, human tissue, or identifiable personal data were collected for this study; therefore, no additional ethics approval or consent to participate was required.

## Consent

The authors have nothing to report.

## Conflicts of Interest

The authors declare no conflicts of interest.

## Supporting Information

Additional supporting information can be found online in the Supporting Information section.

## Supporting information


**Supporting Information 1** Table S1 provides the completed STROBE‐MR checklist.

**Supporting Information 2** Table S2 lists the primer sequences used in the experimental validation.

**Supporting Information 3** Table S3 provides the genetic instrumental variables used for the Mendelian randomization analysis of ferroptosis‐related proteins and pancreatic cancer.

**Supporting Information 4** Table S4 summarizes the full Mendelian randomization results for ferroptosis‐related proteins and pancreatic cancer risk.

**Supporting Information 5** Table S5 provides the proteome‐wide Mendelian randomization results identifying proteins associated with pancreatic cancer risk.

**Supporting Information 6** Figure S1 shows the densitometric quantification of western blot bands related to CLEC4G expression, CLEC4G knockdown efficiency, and CLEC4G/CTSB protein changes after CLEC4G knockdown.

## Data Availability

The proteomic pQTL summary statistics analyzed in this study are available from the deCODE genetics summary data portal (https://www.decode.com/summarydata/) [14] and the UKB‐PPP resources (https://registry.opendata.aws/ukbppp/) [26]. Pancreatic cancer GWAS summary statistics were obtained from FinnGen [27] and can be accessed via the FinnGen Access Results portal (https://www.finngen.fi/en/access_results), subject to the FinnGen data access procedures. Additional information and analysis code are available from the corresponding authors upon reasonable request.
