# Supplementary figures and images for: CLEC4G Promotes Pancreatic Cancer Progression by Suppressing Cathepsin B‐Mediated Ferroptosis: Evidence From Mendelian Randomization Study and Experimental Validation
Source: Hum Mutat. 2026 Jun 10;2026:7359548. doi: 10.1155/humu/7359548 (PMC13250469; doi:10.1155/humu/7359548)

## Slide 1
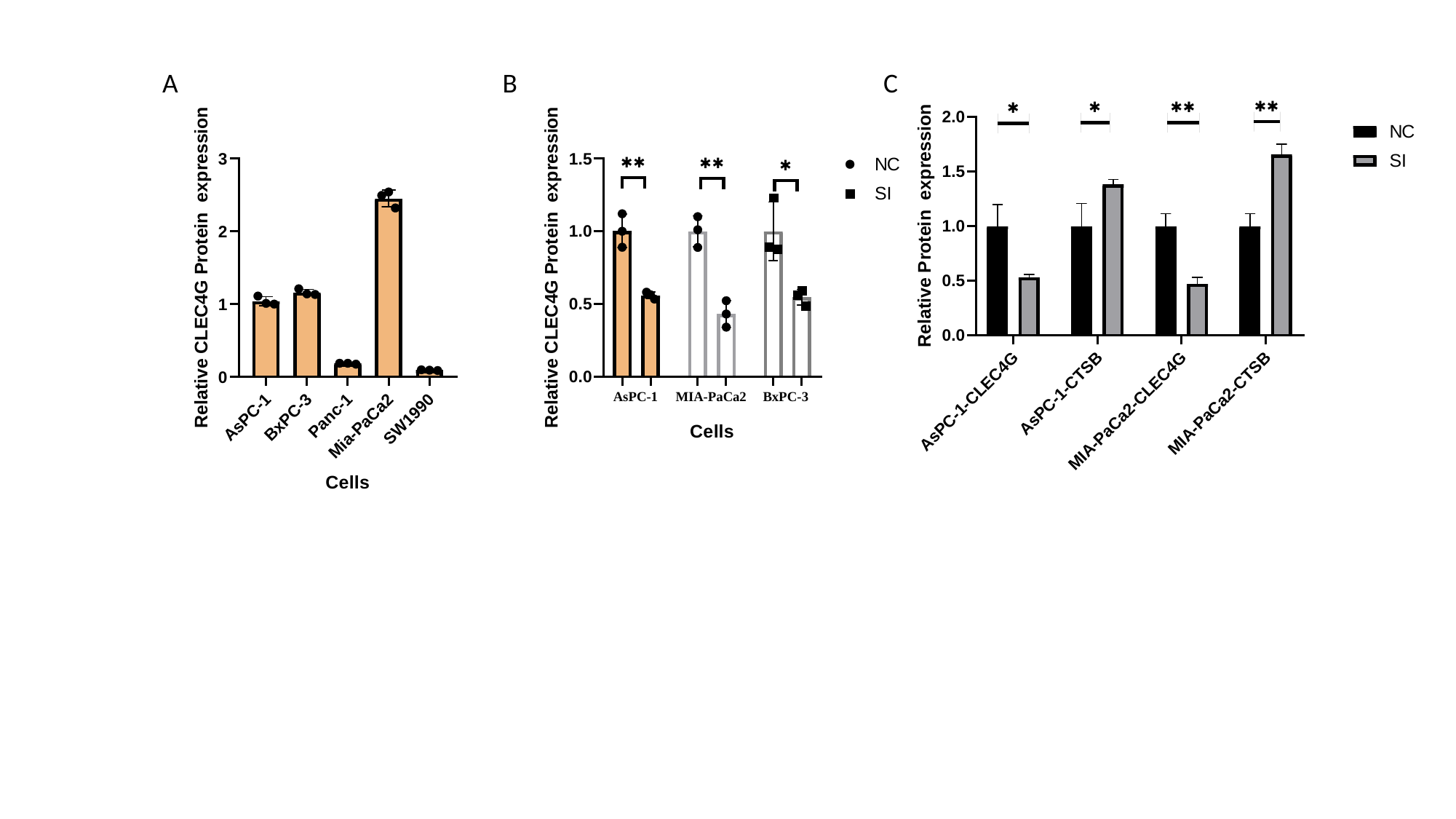

A
B
C

Supplement: Supplementary file 6 — Supporting Information 6 Figure S1 shows the densitometric quantification of western blot bands related to CLEC4G expression, CLEC4G knockdown efficiency, and CLEC4G/CTSB protein changes after CLEC4G knockdown. [file HUMU-2026-7359548-s006.pptx]
